# Knowledge on malignant hyperthermia: as rare as the disease? A nation wide survey

**DOI:** 10.1186/1471-2253-14-S1-A18

**Published:** 2014-08-18

**Authors:** Timothy Wolvetang, Jan Hofland, Hanneke Takkenberg

**Affiliations:** 1 Department of Cardio-thoracic surgery, Erasmus MC, Rotterdam, 3000, Netherlands; 2 Department of cardio-thoracic Anaesthesiology, Erasmus MC, Rotterdam, 3000, Netherlands

## Background

Knowledge on malignant hyperthermia (MH) has expanded vastly during the past decades, but not everyone is up to date on all this newfound knowledge. To assess the current level of knowledge on MH, a survey was performed among Dutch anaesthesia personnel. Research questions were: What is the current general knowledge of anaesthesia personnel about MH; furthermore, do anaesthesiologists (in training) know more than (trainee) nurse anaesthetists; and, does experience with a MH crisis and/or triggerfree anaesthesia result in a better overall knowledge score?

## Materials and methods

The survey consisted of an online questionnaire, responders were recruited via Dutch social media groups for anaesthesia personnel. The survey entailed 12 questions, 3 of which assessed the responders profession, hospital, and whether the participant had previously encountered MH in their practice (i.e. MH crisis and/or triggerfree anaesthesia). The other 9 questions assessed the existing knowledge on MH. The questions covered various important aspects of MH: knowledge on the incidence, triggers, symptoms, prevention, (importance of early) recognition, and treatment. The maximum possible score was 12 points. Crosstabs and one way anova analysis was performed with SPSS Statistical software package version 21 for statistical analysis, P <0,05 was taken to represent significance. Correctness of the answers was assessed in relation to the available literature and EMHG guidelines on the subject.

## Results

A total of 104 (n=104) responders entered the survey, of which 22 were anaesthesiologists; 20 residents; 42 nurse anaesthetists; 17 trainee nurse anaesthetists; 3 did not specify their profession. Among responders 52 subjects had no previous experience with MH in practice, as opposed to 51 who did have experience (1 subject did not specify), the latter group had a significantly higher knowledge score. Results of the knowledge questions and total knowledge scores are shown in table 1.

**Table 1:** Percent correct answers per question and total score, * P <0.05 between groups

Question subject	Total correct	Anaesthesiolo-gists	Residents	Nurse Anaesthetist	Trainee Nurse Anaesthetist
Prevalence	4.8%	9.1%	0.0%	7.1%	0.0%
Prevention (how long should ventilator be flushed with O2)	22.8%	27.3%	25.0%	23.8%	11.8%
Importance of recognition of early symptoms	35.6%	63.6%*	30.0%*	23.8%*	35.3%*
Recognition of tachycardia as early symptom	60.4%	68.2%	65.0%	50.0%	70.6%
Basic triggering medication	74.3%	77.3%	85.0%	66.7%	76.5%
Advanced triggering medication	1,0%	0.0%	0.0%	2.4%	0.0%
Stress as trigger	64.4%	77.3%	70.0%	64.3%	41.2%
Cardiopulmonary bypass as trigger	32.7%	50.0%*	50.0%*	19.0%*	23.5%*
Basic knowledge on later symptoms	30.7%	40.9%*	50.0%*	16.7%*	29.4%*
Advanced knowledge of later symptoms	10.9%	18.2%	15%	2.4%	17.6%
Dosage of Dantrolene IV	38.6%	45.5%	55.0%	23.8%	47.1%
Triggering time of MH	35.6%	59.1%*	35.0%*	23.8%*	35.3%*
Total score (average, max 12)	3.51	4.68*	4.15*	2.74*	3.18*

## Conclusions

Knowledge on MH is not quite as rare as the disease but certainly needs improvement as evidenced by this survey. Anaesthesiologists and residents have significantly better knowledge than (trainee) nurse anaesthetists. Yet the highest average knowledge score of 4.68 out of a maximum of 12 points is disappointing reflecting insufficient basic knowledge on MH. These observations call for improved knowledge dissemination of this rare but very dangerous complication of anaesthesia. Means by which this might be achieved is simulation education as anaesthesia personnel with experience have a significantly better knowledge score.

